# Cellulolytic and Xylanolytic Enzymes from Yeasts: Properties and Industrial Applications

**DOI:** 10.3390/molecules27123783

**Published:** 2022-06-12

**Authors:** Muhammad Sohail, Noora Barzkar, Philippe Michaud, Saeid Tamadoni Jahromi, Olga Babich, Stanislav Sukhikh, Rakesh Das, Reza Nahavandi

**Affiliations:** 1 Department of Microbiology, University of Karachi, Karachi 75270, Pakistan; msohail@uok.edu.pk; 2 Department of Marine Biology, Faculty of Marine Science and Technology, University of Hormozgan, Bandar Abbas 3995, Iran; 3 Institute Pascal, Université Clermont Auvergne, CNRS, Clermont Auvergne INP, F-63000 Clermont-Ferrand, France; philippe.michaud@uca.fr; 4 Persian Gulf and Oman Sea Ecology Research Center, Iranian Fisheries Sciences Research Institute, Agricultural Research Education and Extension Organization (AREEO), Bandar Abbas 3995, Iran; 5 Institute of Living Systems, Immanuel Kant Baltic Federal University, 236016 Kaliningrad, Russia; olich.43@mail.ru (O.B.); stas-asp@mail.ru (S.S.); 6 Department of Paraclinical Sciences, Faculty of Veterinary Medicine, Norwegian University of Life Sciences (NMBU), 1433 Aas, Norway; rdascifa@gmail.com; 7 Animal Science Research Institute of Iran (ASRI), Agricultural Research, Education and Extension Organization (AREEO), Karaj 8361, Iran; rezanahavandi91@gmail.com

**Keywords:** cellulose, cellulases, xylan, xylanases, yeast, applications

## Abstract

Lignocellulose, the main component of plant cell walls, comprises polyaromatic lignin and fermentable materials, cellulose and hemicellulose. It is a plentiful and renewable feedstock for chemicals and energy. It can serve as a raw material for the production of various value-added products, including cellulase and xylanase. Cellulase is essentially required in lignocellulose-based biorefineries and is applied in many commercial processes. Likewise, xylanases are industrially important enzymes applied in papermaking and in the manufacture of prebiotics and pharmaceuticals. Owing to the widespread application of these enzymes, many prokaryotes and eukaryotes have been exploited to produce cellulase and xylanases in good yields, yet yeasts have rarely been explored for their plant-cell-wall-degrading activities. This review is focused on summarizing reports about cellulolytic and xylanolytic yeasts, their properties, and their biotechnological applications.

## 1. Introduction

The diversity of microorganisms in natural habitats, particularly with harsh environmental or chemical conditions, offers vast opportunities for exploration, as these habitats are the source of novel strains that can produce industrially important biomolecules including enzymes [1]. Amongst various groups of microorganisms, the enzymatic potential of yeasts remains less explored than their bacterial and mold counterparts [2]. Yet, yeasts provide several advantages as an enzyme producer, particularly for cellulases and xylanases, as yeast is considered a model eukaryotic group that can be cultivated in a relatively shorter period than molds and are easy to manipulate genetically [3]. Moreover, yeast is preferred over bacteria for fermentative production owing to the easier downstream processes and the tolerance of yeast to bacterial or viral contamination [4].

**Figure 1:**
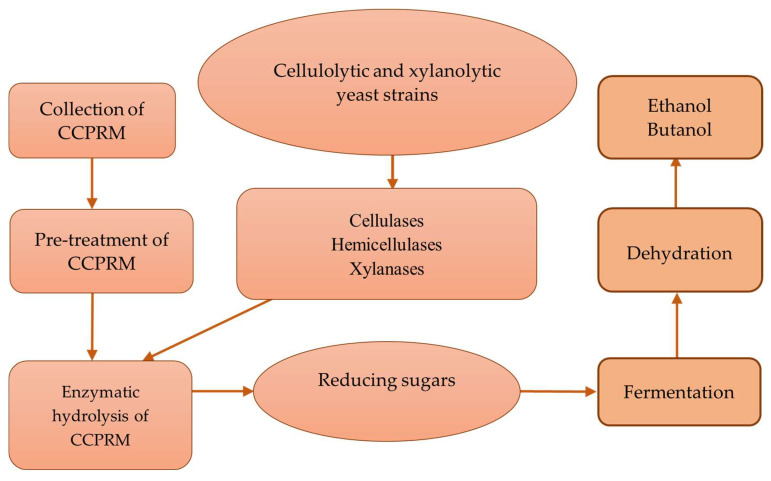
Scheme for producing biofuels from CCPRM using cellulolytic and xylanolytic yeasts.

Cellulose, the world’s most abundant biopolymer and a huge feedstock of chemicals and energy [5], is hydrolyzed into oligo-, di- and monosaccharides by the activity of endoglucanase, exoglucanases and β-glucosidase, which are collectively referred to as cellulase [6]. Cellulases have received enormous attention considering their wide range of industrial applications [7]. Glucose and other simple sugars released by the action of cellulase on cellulose can be utilized as a low-cost fermentation substrate to produce ethanol and other value-added products. The ability of the yeast to withstand environmental conditions, particularly acidic media, renders it a candid organism for biofuel production. Cellulosic fuel is yet available commercially on large scale; however, it remains a topic of extensive research globally. Therefore, cellulolytic yeasts are in high demand, yet few cellulolytic yeast strains have been reported [8]. Figure 1 depicts a scheme for producing biofuels (ethanol and butanol) from cellulose-containing plant raw materials (CCPRM) using cellulolytic and xylanolytic yeasts.

In plant cell walls, hemicellulose is also present in large quantities, along with cellulose. Xylan is the most abundant hemicellulose and is degraded by the activity of xylanases into heterogenous mixtures of sugars. Endo-β-xylanase (EC 3.2.1.8) releases xylooligosaccharides from the xylan backbone; the product of this enzyme is taken up by β-xylosidase (EC 3.2.1.37) to release D-xylose [9,10]. Xylanases also find an array of applications with the supplementation of cellulase or applied as cellulase-free preparation. Indeed, xylanases are applied in many food-related processes such as juice clarification, the extraction of oils and starch from plant materials and to improve silage and feed quality [11,12]. The paper and pulp industry particularly requires cellulase-free xylanase so that the quality of the product is not affected during pulping and/or bleaching [13]. There is a long list of xylanolytic bacterial and mold species with very few yeasts that can utilize xylan as a sole carbon source [14]. 

Here, we summarize recent studies and hypotheses regarding the exploration of cellulases and xylanases from yeasts, emphasizing their biochemical properties and their potential applications.

## 2. Source, Composition and Physico-Chemical Properties of Cellulose

Cellulose, the most abundant polymeric substrate in nature, is the major constituent of plant cell walls and is hence considered a renewable feedstock of energy and chemicals [15,16,17]. Intra-molecular hydrogen bonds amongst the monomers and chains lead to the formation of fibrils and fibers, and in turn, the structure becomes crystalline or semi-crystalline and unusually aggregates in aqueous environments [18,19,20]. Along with the other constituents, it is primarily responsible for the structural integrity of the plant cell wall. 

Wood tissues (40–60%), flax fibers (60–85%), cotton (95–98%), cotton wool and filter paper (up to 90%) all contain significant percentages of cellulose. This cellulose, as the main component of the plant cell structure, is formed in the process of photosynthesis [15] by the cellulose synthase complex present in plasma membrane [20]. It is a condensation polymer of linearly arranged β-1,4-linked glucose molecules whose chains interact with each other through hydrogen bonding [20]. The rigid chain structure and symmetrical arrangement of polar and nonpolar molecules is a hallmark of cellulose among plant polysaccharides [19]. The arrangement of chains of glucose in plants gives rise to the formation of cellulose microfibrils with a lateral size of 3–10 nm [20]. 

In principle, four polymorphs of cellulose (I–IV) have been identified by using X-ray diffraction. Cellulose I, being naturally available is the most dominant form and can further be categorize as α and β according to the subtle differences that can be visualized by NMR [21]. Cellulose obtained from microbial sources is Iα, while plant cellulose is Iβ type [22,23]. ‘Regenerated cellulose’ or cellulose II is irreversibly obtained from cellulose I through washing and recrystallization in sodium hydroxide [21]. Cellulose II is used to prepare yarn or other similar fibers. The treatment of cellulose I or II using ammonia or amine yields cellulose III, while the glycerol treatment of cellulose II at high temperatures produces cellulose IV. X-ray diffraction patterns have revealed similarities between cellulose I and IV [22]. 

Industrially, cellulose is produced by boiling wood chips (pulping) at pulp mills that are part of larger industrial complexes. Several pulping methods are distinguished based on the type of reagents used [24], including sodium hydrogen sulfite (sulfite method), sodium hydroxide (natron method) and a combination of sodium hydroxide and sodium sulfide (sulfate method). 

Various impurities, such as lignin and hemicelluloses, are present in the technical cellulose obtained after cooking. If cellulose is to be used in chemical processing (for example, to produce artificial fibers), it must be refined, which involves removing hemicelluloses with a cold or hot alkali solution [18]. Finally, cellulose is bleached to remove residual lignin and make it whiter. 

Synthetic cellulose should be acknowledged as a potential research topic. It is produced by the polymerization of an aqueous solution of glucose with a concentration from 20 to 40% by weight in the presence of tungsten–vanadium heteropoly acid.

Several types of cellulose were investigated in a study [20], including synthetic cellulose (SC), cotton high viscosity cellulose (CC), and hemp cellulose (HC). The characteristics of several types of cellulose are shown in Table 1.

Crystalline cellulose properties are primarily determined by the properties and structure of the crystals, as well as the presence of various impurities [20].

SC and HC plant celluloses are characterized by a monoclinic cell, according to published data on interplanar distances. However, there are differences in the values of interplanar distances and in the packing of the crystal lattice. The observed changes in the structural organization are directly related to the size of the crystals.

Thus, the size of SC cellulose crystals is 62 A, the degree of crystallinity is 89.4 %, and the degree of polymerization is 1250, which is significantly larger than the size of SC and HC cellulose crystals, which are both 57 A. It was found that, for given crystal sizes, SC and HC celluloses are characterized by a degree of crystallinity of 88.6 and a degree of polymerization of 3140 and 760, respectively [20]. The established differences in the structure of the crystal lattice affect the physicochemical properties of plant and synthetic celluloses [20] and determine the area of their application.

It was noted that, due to its structure, SC cellulose has a high degree of crystallinity and density, which leads to a high specific surface area and a unique ability to form stable thixotropic hydrogels [20].

Synthetic cellulose has a higher asymmetry index of the shape of the absorption band of stretching vibrations of hydroxyl groups than natural analogs, as well as a more uniform distribution of intramolecular and intermolecular hydrogen bonds [20]. 

Cotton cellulose crystallite’s size in the transverse direction is larger than that of synthetic cellulose crystallite, whereas the ratio of the intensity of transverse reflections and the value of the monoclinic angle are like synthetic cellulose parameters. Thermal analysis and inductively coupled plasma mass spectrometry confirmed the high purity and molecular homogeneity of plant cellulose. The structural differences revealed in plant cellulose allowed the production of cellulose nitrate that is not inferior in quality to synthetic cellulose processing products [20].

Cellulose in the form of plant material has remained in use by mankind for many years. In antiquity, it was used as a building material and low-grade fuel, while nowadays, its versatile derivatives find various industrial and commercial applications [27]. Some of its derivatized forms were prepared even before its complete structural elucidation. Nonetheless, nitrocellulose and cellulose acetate are popularly used cellulosic compounds, while the preparation of nanocellulose has shown prospects in industrial applications [28,29]. The high amount of cellulose in some fibers including ramie, hemp and jute (60–90%) renders them suitable for textile industries [29]. Wood chips are also enriched with cellulose and are applied in papermaking. 

Recent advancements in polymer technology have included the application of cellulose along with some other industrial materials to form new chemicals, such as carbon nanotubes, conductive nanocomposite complexes that are applied in various electronic devices and sensors [30]. 

## 3. Classification and Mechanism of Cellulolytic Enzymes

Based on their activity on cellulose, cellulases can be divided into three main types: endoglucanases (EG), which cleave the glucose chain internally and randomly, so oligosaccharides of shorter length are released; exoglucanases, which attack the ends of the chain and reduce its length; and β-glucosidases (BGL), which hydrolyze cellobiose to glucose [6]. According to the CAZy database, these cellulases belong to different glycosyl hydrolase (GH) families [31]. The three enzymes, nonetheless, act in synergy as EG exposes more reducing ends with variable chain length, which can be taken up by exoglucanase (particularly cellobiohydrolase, CBH). Exoglucanase further reduces the degree of polymerization to produce disaccharide or shorter oligosaccharides that act as substrates for BGL [32]. EG is reportedly active towards amorphous and crystalline cellulose [33]. Besides these, some other accessory enzymes are also produced, including cellobiose phosphorylase or cellobiase, cellodextrin phosphorylase and cellobiose epimerase [34]. Swollenin and lytic polysaccharide monooxygenases are the other accessory enzymes with broad specificity [35]. 

Studies deciphering cellulase mechanisms have mainly been carried out on cellulases from bacteria or molds, and hence, the distinct features of yeast cellulases, if there are any, are yet to be explored. Commercially, cellulase preparation (or exoproteome) from *Trichoderma reesei* is widely applied in industrial processes. This organism is well-studied, and its cellulases have been characterized extensively. It secretes two CBH (that constitute almost 70% of the total proteins), three different EG and one each of lytic polysaccharide monooxygenase and BGL [36,37]. The role of CBH in the degradation of type I crystalline cellulose is well-established; indeed, the saccharification is enhanced when this crystalline cellulose is disrupted to other allomorphs [38,39]. 

Vertebrates do not elaborate enzymes to degrade plant cell wall polysaccharides; hence, they exploit the presence of symbiotic microorganisms present in their hindgut. The symbionts can be categorized as pregastric or postgastric depending on the presence of the habitat to the proximal or distal end of the gut. Usually, postgastric presence benefits the host by providing short-chain fatty acids, while in other cases, the animal can utilize the microbial cell and its metabolites. Invertebrates usually do not harbor such symbiosis; rather, they excrete their own cellulolytic enzymes. However, termites in the groups *Hypermastigida*, *Trichomonadida* and *Oxymanidida* carry symbiotic cellulolytic microorganisms in their enlarged hindgut, which is termed a paunch [40]. 

As far as machinery involved in the degradation of cellulose is concerned, aerobic and anaerobic bacteria differ greatly in the way in which they hydrolyze this polymer. In aerobes, individual proteins (6–10) are released, and some of them can have a carbohydrate binding domain (CBM) linked through a flexible linker peptide to the catalytic domain (CD). These and some other peptides can act synergistically, particularly to degrade crystalline cellulose, and the combined action is approximately 15 times more efficient than that of a single peptide. In many aerobes, the cellulase secretions contain one type of exocellulase and different types of endocellulases. Yet, exocellulase comprises more than half of the total proteins in the enzyme preparations [41,42].

In anaerobes, cellulase activity resides in multienzyme complexes, cellulosomes, with a molecular weight exceeding 10^6^ Da [43]. Cellulosomes are usually bound to the outer layer of the cellular envelope. CBM is not present in all the proteins of cellulosomes; however, the scaffoldin protein that has multiple cohesin domains harbors a CBM of family 3 [44,45]. The component enzymes of cellulosomes are joined with cohesin through small dockerin domains [46]. A flexible linker peptide joins the C-terminus of dockerin domain to CD. The exocellulases in bacterial cellulosomes attack the reducing and non-reducing end of cellulose, whereas the endocellulases attack inner linkages of the molecule [47]. Hence, the proteins in cellulosomes exhibit a great degree of synergy. Cellulosomes, in at least some organisms, can be more complex with the catalytic potential to degrade different plant cell wall components [48]. For instance, *Clostridium thermocellum* contains at least 72 different cellulosomal genes [49]. Furthermore, it is still unclear if the organization of proteins in cellulosomes is a random process, or if it is a directed self-assembly process. All the dockerins exhibit the same affinity towards cohesin; therefore, it is likely that the process is a randomized self-assembly process in each organism. Although the proximity of different cellulolytic proteins in a cellulosome provides an advantage in cellulose hydrolysis, the large area of the structure limits its access to the substrate present in small pores [50]. 

Apart from the two mechanisms of cellulase degradation discussed above, *Cytophaga hutchinsonii* and *Fibrobacter succinogenes* degrade cellulose through an entirely different mechanism [51,52,53]. In *C.* *hutchinsonii*, the genes encoding dockerin domains are not present, while very few CBM encoding genes are found. Yet, there are many EG-encoding genes. Therefore, this organism lacks processive cellulases, which are commonly present in most cellulolytic organisms [54]. Hence, the lifestyle of this organism on cellulose does not match with the known aerobic and anaerobic cellulolytic bacteria. Likewise, the genome elucidation of *F. succinogenes*, a ruminal bacterium, does not indicate the presence of processive cellulases [55]. The cloning of its EG revealed the presence of proteins that can bind with the cellulose. *F. succinogenes* grows efficiently on cellulosic substrates, yet it does not express any dockerin domain or scaffoldin gene [56]. Therefore, the cellulose degradation by *C.* *hutchinsonii* and *F. succinogenes* does not follow the mechanisms through which aerobic and anerobic bacteria work. It is proposed that perhaps the two organisms catalyze cellulose degradation in a similar way as *Bacteroides thetaiotaomicron* degrades starch, where starch initially binds to an outer membrane protein complex, and then individual starch molecules are transported to periplasmic space where these are degraded by specific amylolytic proteins [57]. Hence, the processive proteins do not play any role in starch degradation. Yet, it is not clear how the outer membrane proteins bind to the substrate and then transport the individual molecules. Nonetheless, the exploration of this mechanism will aid in designing new cellulose-degrading proteins and complexes with a higher rate of catalysis [50].

## 4. Cellulolytic Microorganisms

The genes encoding cellulases are widely distributed in different groups of microorganisms, but molds are usually applied for large scale production due to the secretion of higher titers of cellulases [34]. Industrial production demands a different set of tolerance to the environmental and chemical factors; therefore, the exploration of novel species of cellulolytic organism is continuously occurring [58]. Generally, microbial strains utilize crystalline or soluble cellulosic substrates, which are comparatively costly; therefore, agricultural and forestry residues have also been reported as cellulosic substrates [59,60,61]. 

Cellulolytic microorganisms depend on carbohydrates to fulfill their energy demands, and very little or no energy is obtained from lipids and proteins [32]. Therefore, higher titers of cellulases are produced when these are cultivated on cellulosic substrates. For instance, *Penicillium* sp. has been found to release copious amount of cellulase in rice-straw-containing medium. The enzyme from this organism performed optimally at 65 °C and pH 4–5 [62]. Annamalai et al. [63] obtained 4040.45 U mL^−1^ of cellulase from marine *Bacillus carboniphilus* CAS 3 when cultivated at 50 °C on a medium containing rice bran and yeast extract (in approximately a 3:1 ratio) with an initial pH of 9.0 [63]. A combination of *Trichoderma reesei*, it’s mutants and *Aspergillus phoenicis* QM329 was studied, and enhanced EG and BGL activities were produced from a co-culture of parent *T. reesei* with *A. phoenicis* on bagasse [64]. While comparing the productivity of cellulase by *Trichoderma* sp. and *A. niger* on municipal solid waste (MSW), Gautam et al. (2011) concluded that 4% MSW was optimal, and *Trichoderma* yielded higher titers of EG and BGL than did *A. niger* [65]. Liu et al. (2011) adopted a strategy to obtain cellulosic ethanol from *A. fumigatus* Z5 and cultivated the strain under solid-state fermentation (SSF) of agricultural residues. The authors reported 0.112 g bioethanol g^−1^ dry substrate, along with the production of thermostable cellulase [66]. 

In another study, researchers attempted to replace simple sugars (glucose, fructose, maltose, and sucrose) from the production medium of *B. amyloliquefaciens* DL-3 and found that the strain produced 102 U mL^−1^ of cellulase in the medium containing 2% rice hull and 0.25% peptone [67]. Likewise, Waghmare et al. [68] reported sugarcane barbojo and grass powder as the most suitable carbon sources for cellulase production from *Klebsiella* sp. PRW-1 in comparison to the other agricultural residues including straws from corn and paddy field, sugarcane bagasse, sorghum husks and grass powder [68]. In some other studies, potato peels and banana wastes have been stated as promising sources of the cellulase production from *A. niger* and *Pleurotus* sp., respectively. Banana peels have also been used as substrate in the growth medium of *B. subtilis* (CBTK 106), and improved cellulase production was reported [69]. Similarly, Shah and colleagues [70] evaluated the efficacy of banana agricultural waste in the fermentation medium of *Aspergillus* sp. MPS-002 and *Phyllosticta* sp. MPS-001 to obtain cellulolytic enzymes [70]. In yet another study, corncob was used to produce thermostable cellulase from *Sporothrix carnis*, and a titer of 285.7 U mL^−1^ was obtained within 96 h at 80 °C using 2.5% inoculum at pH 6.0 [71].

The approach of the design of experiments, through statistical tools, has also been applied to enhance cellulase production. Alam et al. [72] obtained a 1.5-fold enhancement in the cellulase production from *T. harzianum* by adopting two-level factorial design and by using sludge from wastewater treatment and wheat flour [72]. The process engineering of a fed batch culture by another group of workers [73] also resulted in an improvement in cellulase production from *T. reesei* RUT C3 and 90.3 FPU mL^−1^ of cellulase in 144 h, and a productivity of 627.1 FPU L^−1^ h^−1^ was achieved by controlling glucose flow at low level. 

Studies on *B. halodurans* CAS 1 revealed that the strain optimally produced a halotolerant and thermo-alkaline cellulase at 60 °C and pH 9.0 when grown on agricultural residues. The enzyme activity was sensitive to EDTA and PMSF, indicating its nature as a metalloenzyme and having serine residue in its catalytic site [74]. 

## 5. Cellulolytic Yeasts

Cellulases are popular industrial enzymes that share approximately 20% of the global enzyme market with pectinase and hemicellulases [7]. Glucose and other simple sugars released by the action of cellulase on cellulose can be utilized as a low-cost fermentation substrate to produce ethanol and other value-added products. 

Naturally, very few yeasts demonstrate a cellulose-degrading ability. For instance, Jiménez et al. [75] isolated 51 yeast species from decaying wood; the species belonged to the genera *Aureobasidium*, *Candida*, *Filobasidium*, *Kluyveromyces*, *Pichia* and *Trichosporon*. Only *Aureobasidium microstictum* and *Trichosporon pullulans* exhibited cellulase activity when grown on carboxymethyl cellulose (CMC) or wood chips [75] (Table 2). Later, a report claimed to be the first of its kind where the purification of endo-β-glucanase from a psychrophilic yeast species, *Rhodotorula glutinis*, was described [76]. In their studies on the habitat of *Forcipomyia taiwana* (biting midge), Chen et al. [77] concluded that *Aureobasidium* sp. is a predominantly found cellulolytic yeast in that environment. 

The use of yeast, such as the strains of *Dendrobium spathilingue*, *Moesziomyces* sp., *Candida easanensis* and *Saccharomyces* species, for the hydrolysis of crude lignocellulosic substrates has also been reported by a few researchers (Table 2). Recently, Shariq and Sohail [78] reported the co-production of cellulase and xylanase from an indigenous strain of *Candida tropicalis* MK-118 [78].

Besides terrestrial yeasts, marine yeasts have also been studied extensively for their cellulolytic activity. For instance, Rong et al. [79] studied the yeast ecology of surface seawater of sea saltern in the Yellow sea (China), with a particular emphasis on the screening of yeast for cellulase production [79]. Likewise, marine species of *Aureobasidium pullulans*, black yeast, has also been reported for EG and exoglucanase production [80]. Despite the biotechnological importance of marine-derived enzymes [81,82,83], the biotechnological application of marine-derived cellulolytic yeast has yet to be established.

**Table 2:** Examples of few reported cellulolytic yeasts.

Origin	Source	Isolated From	Strains	Reference
Terrestrial	Bali National Park, Indonesia	Dendrobium flower	Isolates D.2.7 and W.3.8	[2]
	-	Decayed wood	*Aureobasidium microstictum*, *Trichosporon pullulans*	[75]
	-	Enriched soil sample	*Candida tropicalis* (MK-118)	[78]
	Laboratory of Bioprocesses and Sustainable Products (LBIOS–UNESP, Rio Claro, SP, Brazil)	-	*Aureobasidium pullulans* LB83	[84]
	Mushroom farm in Yala Local Government Area of Cross River State, Nigeria	Soil samples	*Saccharomyces cerevisiae* SCPW 17	[85]
	Southeast Sulawesi, Indonesia	Leaf and leaf litter samples	*Candida* sp. *Sporodiobolus* sp. *Pichia* sp. *Pseudozyma* sp. *Sporobolomyces* sp.	[86]
	Onam-ri, Gyeonggi Province, South Korea	Gut of Grasshopper	*Moesziomyces* sp.	[87]
	Toledo River, Parana (PR), Brazil	Water samples	*Apiotrichum mycotoxinivorans*	[88]
	decaying leaves, wood and ant nests from Brazil	Decaying leaves, wood and ant nest	*Trichosporon laibachii* MG270406-1A14 strain	[89]
	-	-	*Trichosporon* sp.	[90]
	Eastern Ghats region of Thandikudi, Tamil Nadu, India	Forest soil samples	*Trichosporon asahii*	[91]
	-	-	*Saccharomyces cerevisiae*	[92]
	Five different regions around the local area, Jodhpur, India	Soil rich in cellulosic waste	*Cystobasidium oligophagum*	[93]
	Batanta Island Raja Ampat, West Papua Province, Indonesia	Multiple soil cores (15 cm depth by 2 cm diameter)	*Sporobolomyces poonsookiae*, *Rhodosporidium paludigenum*, and *Cryptococcus flavescens*	[94]
	RongKho forest, Ubon Ratchathani University, Thailand	Soil, tree barks and insect frass	*Candida* sp. 05-7-186T, *Candida easanensis* and *Candida* sp. ST-390	[95]
	Gunung Halimun National Park, Indonesia	Soil, and rhizosphere soil	*Debaryomyces* sp., *Rhodotorula* sp., *Pichia* sp. and *Candida* sp.	[96]
	King George Island, sub-Antarctic region	Soil samples	*Leucosporidiella fragaria* and *Mrakia* sp.	[97]
Marine	Yellow Sea, China	Sea saltern	*Aureobasidium pullulans* 98	[79]
	-	-	*Aureobasidium pullulans* 98	[98]

## 6. Basic Properties of Cellulolytic Yeasts

Although cellulolytic yeasts have not been widely reported, researchers have obtained promising data. While exploring the habitat of RongKho forest, Ubon Ratchathani University, Thongekkaew and Kongsanthia, [95] isolated 82 yeast species, out of which three strains of *Candida* produced comparable titers of cellulase in the range of 0.224–0.238 UmL^−1^. The yeasts were found to grow and release cellulase optimally after 168 h of cultivation in the presence of yeast extract and carboxymethyl cellulose at 30 °C with shaking at 150 rpm [95] (Table 3).

*Cryptococcus laurentii* isolated from chicory co-produced cellulase (0.1 U mL^−1^ each of CMCase and FPase) and endo-xylanase (2.7 U mL^−1^) [99]. The yeasts isolated from animal guts have also been studied for the optimal parameters affecting the growth and production of cellulase. In this context, a yeast strain isolated from a coprophage, *Gymnopleurus sturmi*, was found to be a promising cellulolytic strain [90]. The data revealed that the enzyme from this strain is capable of hydrolyzing various cellulosic substrates, including insoluble fibers and filter paper (FP); the enzymatic activity remained unaffected with a change in pH and temperatures from 55 to 70 °C. The enzyme showed better catalysis at a higher substrate loading of 100 mg mL^−1^ of CMC or FP. The highest activities of 0.52 IU mL^−1^ for the CMCase were obtained, while FPase activity needed extended time so that the enzyme could be adsorbed to the substrate [90] (Table 3).

A marine yeast, *Aureobasidium pullulans* 98, originally isolated from the surface seawater, produced 4.51 U mg^−1^ of CMCase and 4.75 U mg^−1^ of FPase. The CMCase from this strain had a molecular weight of 67.0 kDa [98]. Further studies by Rong et al. [79] on this CMCase (from *A. pullulans* 98) showed that the enzyme is activated by Na^+^, Mg^2+^, Ca^2+^, K^+^, Fe^2+^ and Cu^2+^, while the presence of Fe^3+^, Ba^2+^, Zn^2+^, Mn^2+^ and Ag^+^ inhibits its activity. Studies on the kinetic parameters of this enzyme presented 4.7 mg mL^−1^ of *K*_m_ and 0.57 µmol L^−1^ min^−1^ (mg protein)^−1^ of *V*_max_ [79] (Table 3).

**Table 3:** Some biochemical properties of cellulases isolated from yeasts.

Species	Type of Enzyme	Some of the Biochemical Properties of Cellulase Isolated from Yeasts	References
*Trichosporon* sp.	Endoglucanase and FPase	CMCase worked optimally at 55 °C and pH 5, while for FPase activity, temperature 60 °C and pH 4 to 6 found optimal. Both the enzymes showed best activity when reacted with 100 mg/mL of their respective substrates. FPase required more time to catalyze the reaction, probably due to the time taken for adsorption to the insoluble substrate	[90]
*Aureobasidium pullulans* 98	Endoglucanase	The strain produced a titer of 4.51 CMCase U mg^−1^ of protein. The enzyme has a molecular mass of 67.0 kDa, as determined by SDS PAGE. The enzyme exhibited its maximum catalytic efficiency at 40 °C and pH 5.6, but it did not lose its activity in the buffer of pH 5.0–6.0. Na^+^, Mg^2+^, Ca^2+^, K^+^ Fe^2+^ and Cu^2+^ were found to be activators of the enzyme, while Fe^3+^, Ba^2+^, Zn^2+^, Mn^2+^ and Ag^+^ inhibited its activity. *K*_m_ and *V*_max_ values of the purified enzyme were 4.7 mg mL^−1^ and 0.57 µmol L^−1^ min^−1^ (mg protein)^−1^, respectively.	[79]
*Trichosporon asahii*	Endoglucanase	Under optimum conditions, the strain produced 35.70 U of the cellulase, which was reduced to 23.87 U when CMC in the medium was replaced by Napier biomass. The enzyme preparation saccharified the crude substrate with 33.15% yield in 3 days.	[91]
*Sporobolomyces poonsookiae*, *Rhodosporidium paludigenum*, and *Cryptococcus flavescens*	Endoglucanase	The cellulolytic potential of the three strains *Sporobolomyces poonsookiae* Y08RA07, *Rhodosporidium paludigenum* Y08RA29 and *Cryptococcus flavescens* Y08RA33 was evaluated where *R. paludigenum* appeared promising with an index value of 2.60. The isolate *S. poonsookiae* produced CMCase optimally at pH 8 and 37 °C, while *R. paludigenum* and *C. flavescens* produced the highest CMCase levels at pH 6 and 28 °C. Paper waste appeared a good substrate for *S. poonsookiae*, while bamboo leaf was also utilized by the same substrate by this strain and by *C. flavescens. R. paludigenum*, however, was cultivated on soluble CMC to produce optimal titers of cellulase.	[94]
*Candida* sp. 05-7-186T, *Candida easanensis* and *Candida* sp. ST-390	Endoglucanase	The optimization studies showed that 1.0% CMC causes the most effective induction of the enzyme production by all three strains. An incubation period of 96 h was found suitable for cellulase production by *Candida* sp. 05-7-186T (0.224 UmL^−1^), while *Candida easanensis* (0.238 UmL^−1^ and *Candida* sp. ST-390 (0.26 UmL^−1^) produced highest titers in 120 h.	[95]
*Candida tropicalis* (MK-118)	Endoglucanase and β-glucosidase	This strain co-produced endoglucanase (EG) and β-glucosidase (BGL) at 40 °C but at different pH. Neutral pH favored the EG production, while enhanced BGL titers were obtained in acidic medium. For both the enzyme productions, an inoculum size of 2% was found appropriate, but in the presence of respective inducer i.e., CMC for EG and salicin for BGL.	[78]

## 7. Application of Cellulolytic Yeasts

Microbial cellulolytic enzymes find many applications in various industries from pulp and paper to breweries and from textile to waste management. Hence, many studies on process optimization to improve the economics of cellulase production have been conducted [100].

Lately, the surge of alternate sources to obtain renewable fuels, particularly bioethanol, stimulated the research of cellulolytic microorganisms [77]. In this context, cellulolytic yeasts provide a broad spectrum of application as these can be cultivated on cellulosic substrate with the concomitant production of ethanol either by co-culturing with the ethanologenic strain or by isolating a strain with the cellulolytic and ethanol producing ability. Giese et al. [89] reported the potential of *Trichosporon laibachii* MG270406-1A14 to release higher quantities of reducing sugars and its cultivation on lignocellulosic biomass; the reducing sugars can be utilized as a raw material for second-generation biofuel production.

Interestingly, some yeast strains also exhibit environmental stability features comparable to bacterial strains [77,89]. The production of thermostable cellulase that can withstand temperatures of 70 °C and above has also been reported from a yeast strain [101,102]. Likewise, the cellulases produced by *Trichosporon* sp. could hydrolyze a variety of cellulosic substrates at elevated temperatures and at a wide range of pH [90]. Such enzymes offer advantages because of their longer half-lives, and their reactions become protected from contamination; moreover, a concentrated solution of sugars can be used at higher temperatures.

The continuous use of cellulosic biomass to produce valuable bioethanol is an ideal method, but cellulose hydrolysis is difficult and time-consuming. Cellulosomes, which are multi-enzyme complexes derived from anaerobic bacteria, have the best natural cellulolytic effect. Many researchers are working on the construction of cellulosomes in industrial yeasts. However, due to the size and complexity of cellulosome genes, obtaining cellulosomes remains difficult.

The identification of cellulolytic microorganisms is of great interest for cellulosic biomass hydrolysis. In most cases, research is focused on identifying cellulolytic yeasts and optimizing the activity of cellulase produced by the most effective yeast isolate. The study [90] resulted in the selection of 30 cellulolytic yeast isolates. Enzymes produced by isolates of *Trichosporon* sp. showed the ability to hydrolyze different substrates (carboxymethyl cellulose (CMC), cellulose fiber and filter paper). It was discovered that cellulases produced by the examined yeasts can hydrolyze both soluble and insoluble substrates at high temperatures and over a wide pH range, indicating that lignocellulosic substrates can be used to produce fermentable sugars and bioethanol.

A study [88] focused on the search for yeast with an ability to synthesize hydrolytic enzymes. In all, 75 yeast strains were grown in yeast extract-peptone-dextrose (YPD) medium supplemented with antibacterial agents, and their ability to produce enzymes was tested in the specific media. As a result, 64 yeast species demonstrated the ability to produce enzymes. Six of them had good enzyme scores (three for amylase, two for cellulase, and one for protease). All the isolates were tested positive for at least one hydrolytic enzymatic activity (amylases, cellulases, and proteases), suggesting their metabolic potential. The yeasts *Naganishia diffluens* and *Apiotrichum mycotoxinivorans* were found to have the highest enzymatic activity. These yeasts have shown great promise and can be employed as catalysts in the development of bioethanol and biobutanol technologies for industrial usage [88].

*Kluyveromyces marxianus* was studied for its ability to express the “biggest cellulolytic complex”, with up to 63 enzymes on its cell surface [103]. The transformed yeast demonstrated a better efficiency of cellulose degradation and stimulated the release of much more reducing sugars and ethanol from cellulosic substrates than non-cellulosomal constructions due to the synergistic effect of cellulase from cellulosomes. This cellulosomal complex can also be used for the synthesis of various biopharmaceutical products (for example, astaxanthin and/or morphine), which require several stages of enzymatic conversion [103].

The cellulolytic activity of yeast isolates collected from various fruits and fruit wastes was evaluated in terms of cellulose degradation, reducing sugar release, and bioethanol generation in a study [104]. Experimental isolates CY-3, CY-4, CY-8 and CY-15, together with the reference cellulolytic strain of *S. cerevisiae*, demonstrated the maximum hydrolysis of CMC to form reducing sugars when determined at intervals of 2 to 22 days. The isolates were then tested for combined saccharification and the fermentation of a pretreated lignocellulosic substrate (wheat straw). The concentration of ethanol produced by cellulolytic yeast at the end of fermentation was determined using gas chromatography. The CY-15 strain produced 0.573 g L^−1^ of ethanol, which was on the same level as the reference strain (0.68 g L^−1^). This study presented a technology to produce bioethanol by the simultaneous saccharification and fermentation of wheat straw using native yeasts [104]. Moreover, yeast cellulase has also been applied for fruit juice clarification along with the pectinase [12]. Cellulase aids in the removal of the cell wall component, and pectinase clarifies the juice effectively.

Significant efforts to obtain yeast strains for the hydrolysis of cellulose-containing biomass have resulted in the development of industrially applicable biological methods for bioethanol production. *S. cerevisiae* is a reliable microbial platform widely used in biofuel production due to its compatibility with synthetic biology systems and tools. Critical issues for the efficient microbial conversion of lignocellulosic biomass with engineered *S. cerevisiae* include the heterologous expression of cellulolytic enzymes, the co-fermentation of hexose and pentose sugars and resistance to various stresses. Many engineering strategies for *S. cerevisiae* cellulolytics have been developed, bringing the consolidated bioprocess to an industrial scale [105].

## 8. What Is Xylan?

**Figure 2:**
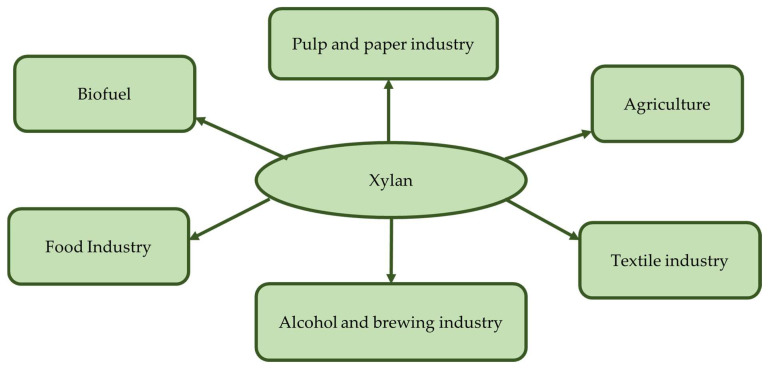
Industries using xylan.

The term ‘hemicellulose’ was coined by Schulze [106] for dilute alkali-extracted fractions from plant materials. Later, it was found that it is not related to cellulose; rather, this is a heterogenous fraction comprising xylan, xyloglucan, glucomannan, galactoglucomannan, arabinogalactan or other heteropolymers [107]. Xylan is the most abundant hemicellulose component, which can constitute approximately 30–35% of the dry weight of the plant cell wall. Data suggest that xylan accounts for more than 30% of the renewable organic compounds on this planet [108,109]. In secondary cell walls, xylan plays an important role to keep the cell wall intact by interacting with cellulose and lignin at the interface between the two [110]. Figure 2 shows the main applications of xylan.

Lignocellulosic biomass, including cellulose, lignin, and hemicellulose in plant secondary cell walls (SCW), is the most abundant source of renewable materials on Earth. Currently, the main lignocellulosic (wood fiber) raw materials for bioproducts are fast-growing woody dicotyledonous plants such as eucalyptus and poplar (cellulose, paper, cellulose, textiles, bioplastics, bioethanol, etc.). The most efficient separation of biomass into three primary components without sacrificing yield is required in the processing of wood to obtain these products. The most abundant hemicellulose, xylan, carries chemical modifications related to the composition and ultrastructure of the SCW and influences the resistance of woody biomass to industrial processing. The importance of xylan properties should be emphasized for the industrial processing of wood fiber and how a deeper understanding of xylan biosynthesis, in particular xylan modification, can lead to new biotechnological approaches to improve the processing efficiency or modification and technological characteristics of xylan [111].

It is known that xylan prevents the hydrolysis of plant biomass during application in bioenergetics [112]. Critical understanding of the composition, structure and biosynthesis of xylan determines the strategy for cell wall degradation in the production of biofuels. The types of xylan epitopes (representing substituted and unsubstituted regions on the xylan backbone composed of β-(1,4)-linked xylose residues) and the strength of their integration into the final wall structure vary during the maturation of the cellulose-containing biomass, according to experimental results and confirmatory in silico analysis [112].

Considering the abundance of xylan, the use of *S. cerevisiae* in its hydrolysis is crucial for the cost-effective production of ethanol from plant biomass [113]. A recombinant strain of *S. cerevisiae* that degrades xylan and assimilates xylose was constructed by co-expressing *T. reesei* xylanase (xyn2), *Aspergillus niger* xylosidase (xlnD), *Scheffersomyces stipitis* xylulose kinase (xyl3) and optimized xylose isomerase (xylA) from *Bacteroides theta*. Under aerobic conditions, the recombinant strain exhibited a complete respiratory regimen, resulting in higher yeast biomass production and hence higher enzyme production during growth on xylose as a carbohydrate source. Under the conditions of oxygen limitation, the strain produced ethanol from xylose with a maximum theoretical yield of approximately 90%. This study is one of the few representing *S. cerevisiae* strains capable of growing on xylan as the sole source of carbohydrates using recombinant enzymes [113].

**Figure 3:**
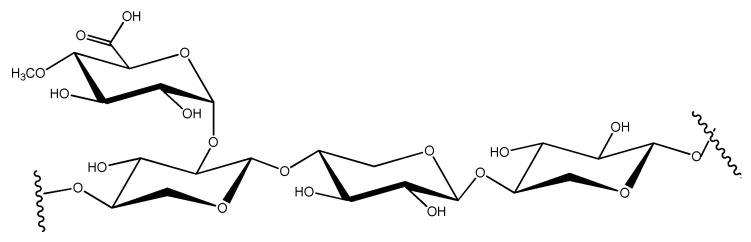
Chemical structure of xylan.

In xylan, a pentose sugar, xylose, polymerizes through β-1,4-linkages to form chains that are branched with different sugars (Figure 3).

Branching occurs at the 0–3 position of xylose by L-arabinofuranose or at the 0–2 position by 4-O-methyl-D-glucronic acid [114]. The amount of xylan and degree of branching in its structure mainly depend on its source [115]. Usually, wood xylan is highly acetylated, which contributes to its solubility. Yet, the studies on microbial degradation of xylan have largely neglected the role of acetylation. In hardwoods, the degree of polymerization (DP) may reach to 150–200 with every 10th xylose residue acetylated with 4-O-methylglucuronic acid (more frequently at position 3 than position 2) [116]. In softwoods, arabino-4-O-methylglucuroxylans are commonly found and linked at position 2. In softwood xylans, α-L-arabinofuranose units are present in place of acetyl groups [117]. Additionally, the chains of xylan in softwoods are smaller (DP 70–130) with lesser branching than hardwood xylans [118]. Generally, a ratio of 1:8 between arabinose to xylose is described to characterize softwoods. The structure of xylan becomes more complex when α-L-arabinofuranosyl units are further substituted at 0–5 by a combination of ferulic and p-coumaric acid (i.e., hydroxycinnamates) [119]. The substituted units are commonly 4-O-methylglucuronic acid cross-links with the aliphatic moiety in lignin to stabilize the structure. Particularly in the secondary cell wall, lignin and xylan are interconnected by a glycosidic bond between xylopyranosyl and p-coumaric acid or by an ester bond between arabinofuranosyl and p-coumaric acid or ferulic acid [120,121], whereas with polysaccharides including cellulose and pectin, xylan is mainly connected with non-covalent interactions [122,123]. Less frequently, such as in esparto grass [124], tobacco stalk [125] and guar seed husk [126], homoxylan is present without any substituent. In marine algae, xylose residues are unusually linked with β-1,3-linkages [127], while in sea weeds, a mix of β-1,3 and β-1,4 linkages are found [128].

The elucidation of xylan structure at molecular level through X-ray diffraction and conformational analysis revealed that the polymer acquires a three-fold, left-handed helical structure [123]. The structure resembles cellulose and mannan when a stereochemical flat ribbon-like formation is observed. The type and degree of branching varies with the variation in the source of xylan; therefore, the structure of xylan exhibits modification, although very little is known about it [129].

## 9. Xylan Degradation

The degradation of hemicellulose in general, and xylan in particular, requires concerted action of the enzymes of different catalytic potential, owing to the heterogenous nature of the substrate [110]. The enzymes termed as xylanases directly attack xylan, along with auxiliary enzymes, such as esterases or, particularly, carbohydrate esterases (CE), and oxidative enzymes i.e., lytic polysaccharide monooxygenases (LPMOs) [130,131]. Xylanases (EC 3.2.1.8) belong to several glycosyl hydrolase (GH) families including family 5, 8, 9, 10, 11, 16, 30, 43, 51 and 98 in the CAZy database (www.cazy.org/, accessed on 13 March 2022). Endoxylanases attack β-1,4-glycosidic linkages internally and release a mixture of xylobiose, xylotriose, xylotetraose, longer and/or branched xylo-oligomers (XOS). Exceptionally, some GH5 enzymes in the subfamily 34 exhibit affinity towards arabinose-substituted xylans [132]. GH11 xylanase activity dwindles in the presence of substituted substrates but remains highly efficient in degrading unsubstituted xylan [133]. Glucuronoxylanases belonging to GH30 depend on the presence of methyl-glucuronic acid for their activity [134]. Lately, oligosaccharide reducing-end xylanases from the GH 8 have been described that catalyze from the reducing ends of xylan or XOs and release either xylooligosaccharides of shorter length or xylose [135,136]. Xylosidases (E.C 3.2.1.37) in the families GH 1, 2, 3, 5, 30, 39, 43, 51, 52, 54, 116 and 120 also produce xylose through its catalysis but, it only works from the non-reducing ends and cannot degrade xylan [137,138].

Alpha-arabinofuranosidases (EC 3.2.1.55) hydrolyze α-L-arabinofuranoside residues in arabinoxylan and are included in the GH families 2, 3, 10, 43, 51, 54 and 62. Usually, these enzymes have specificity to remove the substituent either from mono-substituted or from double-substituted main-chain D-xylopyranosyl motifs, but the enzymes from GH43 and 51 exhibit both specificities [139].

Alpha-glucuronidases (EC 3.2.1.139) release α-(1,2)-D-(4-O-methyl)-glucuronosyl side chains from glucoxylan or arabinoglucoxylans. These enzymes prefer XOs substituted with glucuronic acid (GH67 glucuronidases) or have affinity towards polymeric substrate with glucuronic acid side chains (GH 115). Moreover, the former are generally intracellular or membrane-associated [140,141,142], while the latter are extracellularly produced enzymes [143,144].

CE such as feruloyl esterases, glucuronoyl esterases and acetyl xylan esterases also play important role in xylan degradation. Acetyl xylan esterases (EC 3.1.1.72) found in the CE families 1–7 and 16 in the CAZy database remove acetic acid from the acetylated polysaccharides, thereby increasing the accessibility of backbone-degrading xylanases. Feruloyl esterases (E.C. 3.1.1.73) from CE family 1 break ester bonds between a hydroxycinnamate and arabinoxylan to release a phenolic acid such as ferulic acid or p-coumaric acid. Glucuronoyl esterases (EC3.1.1.-) from CE family 15 cleave the ester bond between lignin aliphatic alcohols and 4-O-methyl-D-glucuronic acid substituents of glucoxylan [145].

LPMOs or auxiliary activity proteins (EC 1.14.99.53-54/56) have shown their prospects in future biomass degradation applications. These proteins work with oxidative mechanism to break glycosidic bonds of polysaccharides in the crystalline and insoluble portion and, ultimately, aid in the completion of the degradation process. Sequence analysis categorized LPMOs into four CAZy families. Few members of the AA9 family are active on hemicellulose, while the members of other families (AA10, AA11 and AA13) work on cellulose, chitin and starch [135]. Like many other high molecular mass polymers, xylan cannot penetrate through the cellular envelope and hence cannot induce the production of xylanases. Therefore, the basal level of the enzyme is constitutively produced to release fragments of xylose, xylobiose and xylooligosaccharides from any available xylan in the environment, and these fragments regulate the genetic expression of xylanases.

## 10. Xylanolytic Microorganisms

As stated earlier, xylan degradation necessitates activities of an array of enzymes, which are produced by various molds [146], actinomycetes [147] and bacteria [147] but less frequently by yeasts. Not surprisingly, many of these xylanolytic organisms serve as a source of thermostable xylanases. Amongst these, many isolates are thermophilic (optimum growth temperature 50–80 °C), while some are hyperthermophilic (grow best at temperature <80 °C). These organisms have been isolated from different environmental niches including hot springs, terrestrial and marine self-solfataric fields and sites of decaying organic matter [148,149,150]. Most of the xylanases produced by these organisms have been categorized into GH 10 and 11, while some have been placed into GH 5, 7, 8 and 43 [151]. *Clostridium thermocellum* [152], *Caldicellulosirutor* sp. [153], *Bacillus stearothermophilus* [154], *Rhodotermus marinus* [155], *Thermoascus aurantiacus* [156] and *Thermotoga* sp. [157] have been reported to produce GH 10 xylanases. One of the well-characterized and highly thermostable GH 10 xylanases is produced by the strain FjSS3-B.1 of *Thermotoga* sp. with an optimum activity at 105 °C and a half-life of 90 min at 95 °C [158]. The sources of GH 11 xylanases include *Caldicoprobacter algeriensis* TH7C1 [159], *Chaetomium thermophilum* [160], *Nonomuraea flexuosa* [160], *Malbranchea cinnamomea* [161], *Paecilomyces varioti* [162] and *Thermomyces lanuginosus* [163]. Contrastingly, the thermostable xylanases from *Dictyoglomus thermophilum* and *Nonomuraea flexuosa* exhibit optimal activity at 85 and 80 °C, respectively. Archaea have also been described for the production of thermostable xylanases such as the strains of *Pyrodictium abyssi* [164], *P. furious* [165], *Sulfolobus solfataricus* [166], *Thermococcus zilligii* [165] and *Thermofilum* sp. [164].

## 11. Xylanolytic Yeasts

Several species of bacteria and molds have been reported for the production of xylanases [167], while xylanolytic yeasts have not frequently been reported [168,169]. Some reports suggest xylan degradation by the strains of *Pichia stiptis* [170], *Cryptococcus*, *Fellomyces* [171] and *Candida* [172]. While working on the microbiota of a lower termite, *Mastotermes darwiniensis*, Handel et al. [173] reported a novel yeast species of *Sugiyamaella* with the ability to produce xylanases. A few species of *Candida* including *Candida* *lignicola*, *Candida* *coipomoensis* and *Candida queiroziae* that were isolated from wood-eating insects were found to have xylose-fermenting potential (Table 4). Due to scarce reports on yeast xylanase, even less data is available on the characterization of these enzymes. Petrescu et al. [174] characterized a cold-adapted endoxylanase from *Cryptococcus adeliensis* (CBS 8351) [174] after its purification [175]. Another study characterized xylanase as cellulase-free enzyme from *Aureobasidium pullulans* CBS 135684 produced on corncob-based media [176]. Contrarily, Shariq and Sohail [172] screened 225 indigenous yeast strains and obtained 84 xylanolytic strains; the unusually high proportion of xylanase-producing strains can be attributed to the selection and enrichment of the samples [172]. *Sugiyamaella xylanicola* and *Saitozyma podzolica* were reported as the most promising xylan-degrading yeasts amongst the screened strains by Morais and coworkers [177], while Otero et al. (2021) found *Cryptococcus* *laurentti* as a potent xylanase producer [178]. In the search of the habitats of unexplored yeasts, Tiwari and colleagues [179] found the gut of wood-feeding termites as a reservoir of unexplored xylanolytic yeasts [179]. Strikingly, there is just one report on the xylanolytic yeast of marine origin [180].

## 12. Basic Properties of Xylanolytic Enzymes from Yeasts

Owing to their industrial applications, particularly in the paper and pulp industry and in biomass conversion, the search for novel xylanases is an ongoing task globally. The exploration of extreme environments is a notable strategy, as the probability of finding strains that can produce temperature- and pH-stable enzymes is higher in such habitats. The optimization of parameters has also been proved to be an effective approach to obtain enzymes with tolerance to a broad range of temperatures and pH [187].

Gomes [175] isolated a psychrophilic yeast, *Cryptococcus adeliae*, from Antarctica, that can produce xylanase and claimed it as the first report of its kind [175]. The authors optimized the culture medium for improved xylanase production by adopting a statistical optimization approach, and an increase by 4.3-fold was obtained. The optimum conditions included medium containing xylan and yeast extract (in 2:1 ratio) with an initial pH of 7.5. The strain produced 400 nkat of xylanase after 168 h. The characterization data presented the xylanase as a thermolabile enzyme with a half-life of 78 min at 35 °C. Although the enzyme was maximally active at 45–50 °C, it lost 71–95% of its activity at 40–50 °C within 5 min, yet it showed good stability at a broad pH range of 4–9, but the maximum activity was obtained at pH 5.0–5.5 [175] (Table 5).

Salah et al. [183] investigated parameters affecting xylanase production by *Pichia membranifaciens* and obtained a higher production after 4 days of fermentation at 35 °C in a medium of pH 7.0. Characterization data showed higher affinity towards beechwood xylan when K*_m_* values were considered. The K*_m_* values of xylanase increased with a decrease in pH from 7 to 4.5 (Table 5).

Morais and colleagues [177] analyzed 200 rotting wood samples for the presence of yeast by using sugarcane bagasse hydrolysate as a medium and isolated 330 yeast strains. Their data presented few promising xylanolytic isolates belong to *Apiotrichum sporotrichoides*, *Aureobasidium pullulans*, *Saitozyma podzolica* and *Sugiyamaella xylanicola*. In this study also, *Sugiyamaella* appeared as an over producer of xylanase that produced optimal titers at 50 °C [177].

In addition to *Sugiyamaella*, *Aureobasidium pullulans* is another frequently reported xylanolytic yeast. The strain CBS 135684 of *A. pullulans* has been studied by Bankeeree et al. [176], where a titer of 4.10 U mL^−1^ was obtained in the medium of corncob as the sole carbon source at room temperature. Moreover, the preparation was found to be cellulase-free with an optimal activity at a high temperature (70 °C). The purified enzyme exhibited activity in a wide pH range of 4.0–10.0; the feature is uncommon among yeasts. The data of negative entropy change at all temperatures indicated a compaction of the protein during denaturation. Interestingly, the supplementation of 0.75 mM sorbitol improved the thermostability of the xylanase preparation (Table 5).

Another yeast, *Moesziomyces antarcticus*, produced 46.6 U mL^−1^ of xylanase titer when cultivated in beechwood xylan. The authors also stated the suitability of Brewery’s spent grain as a medium where the strain PYCC 5535T of *M. aphidis* produced four- to eight-fold higher titers of xylanase than those produced in the presence of xylan or D-xylose. The enzyme exhibited maximum activity at 50 °C and pH 4.5 [188].

In their search for xylanolytic and ethanologenic yeasts, Tiwari et al. [179] investigated different species of termites found in India. Overall, 53 yeast strains were identified, out of which 22 isolates showed xylanolytic activity. Two isolates, namely *Pseudozyma hubeiensis* STAG 1.7 and *Hannaella pagnoccae* STAG 1.14, produced more than 1 IU mL^−1^ of xylanase [179] (Table 5).

**Table 5:** Some biochemical properties of xylanases isolated from yeasts.

Species	Type of Xylanase	Some of the Biochemical Properties of Xylanase Isolated from Yeasts	References
*Candida tropicalis* (MK-118)		The highest titers of xylanase were obtained at 25 °C under neutral pH and in the presence of 1% xylan.	[78]
*Candida tropicalis* MK-160		The strain produced 23.6 IU mL^−1^ of xylanase at 40 °C, in an acidic medium containing 2% xylan. It also produced 5.45% ethanol in glucose-supplemented medium. PO_4_ ^2−^, Co^+2^, Cu^+2^ or Ca^+2^ activated the xylanase activity, while Na^+^, Mg^+2^, Mn^+2^ and K^+^ appeared as inhibitors	[172]
*Aureobasidium pullulans* CBS 135684		The enzyme was purified 17.3-fold with a recovery yield of 13.7%. Its molecular mass was determined through SDS PAGE as 72 kDa. Temperature 70 °C and pH 6.0 were found appropriate for this enzyme’s activity. However, the enzyme retained more than 50% of its activity for 3 h at 50 °C; the addition of 0.75 mM sorbitol improved thermostability up to 10-fold at 70 °C. The enzyme was activated in the presence of Ca^2+^, Co^2+^, and Mg^2+^ and inhibited by Fe^2+^ and Cu^2+^	[176]
*Pichia membranifaciens*		*Pichia membranifaciens* produced the highest titers of 38.8 U mL^−1^ of xylanase in 4 days in a culture medium with pH adjusted to 7.0. Xylanase activity showed maximum activity (42.6 U mL^−1^) at 35 °C in the presence of beechwood xylan. An increase in the K_m_ values of xylanase was noted with a decrease in pH from 7.0 to 4.5.	[183]
*Candida pseudorhagii*		The strain could utilize both xylan and D-xylose but produced more enzyme on xylan (1.73 U mL^−1^) than on D-xylose 0.98 U mL^−1^. The study also reported the identification of four novel strains with the ability to ferment D-xylose and to produce ethanol. Particularly, *C. pseudorhagii* SSA-1542 ^T^ yielded 0.31 g/g of ethanol, with a productivity of 0.31 g L^−1^ h^−1^ and fermentation efficiency of 60.7% in 48 h.	[181]
*Pichia stipitis*		The strain was cultivated under the solid-state fermentation of a corncob and wheat bran mixture and released 5536 U of xylanase per g of substrate. The enzyme was purified, and the molecular weight was determined as 31.6 kDa using SDS PAGE. The optimum temperature and pH were 50 °C and 6.0, respectively. Kinetics parameters were determined as K_m_ 4.52 mg mL^−1^ and V_max_ 9.17 μmol min^−1^ mL^−1^. Xylanase did not lose half of its activity by exposure to 50 °C for 80 min and to pH 5–8 for 60 min. The enzyme remained unaffected in the presence of Cu^2+^ and K^+^.	[184]
*Cryptococcus adeliae*		A statistical approach was used to optimize xylanase production from this strain and an increase by 4.3-fold in the enzyme titers was achieved. The optimized conditions include 12.1 g L^−1^ xylan, 5.1 g L^−1^ yeast extract initial pH of 7.5, temperature 4 °C and incubation time 168 h. A titer of 400nkat of xylanase was obtained in the medium. Although the maximum xylanase titers achieved in presence of xylan, the strain also utilized lignocellulosics. Amongst nitrogenous sources, the organism effectively utilized yeast extract, soymeal, pharmamedia (cotton seed protein) and alburex (potato protein). The crude xylanase remained stable at pH 4–9 for 21 h (at 4°C) but exhibited optimal activity at pH 5.0–5.5. The optimum temperature for the activity was found to be 45–50 °C, but it appeared very thermolabile, with a half-life of 78 min at 35 °C, and at 40–50 °C, it lost 71–95% activity within 5 min.	[175]
*Cryptococcus adeliae*		The study characterized this cold-adaptive enzyme with an apparent molecular weight of 43 kDa as estimated through SDS PAGE. The enzyme had a half-life of 60 min at 30 °C and a melting temperature of 48 °C. The activation energy, Ea, of the psychrophilic enzyme was 47.7 kJ mol^−1^.	[174]
*Pseudozyma hubeiensis*, *Hannaella pagnoccae*, *Papiliotrema mangalensis*, *Kodamaea ohmeri* TS21		The thermotolerance of the strains *Pseudozyma hubeiensis* STAG 1.7 and *Hannaella pagnoccae* STAG 1.14 was evident at 45 °C with the concomitant production of xylanase activities of 1.31 and 1.17 IU, respectively. The other two strains yielded less than 1 IU of xylanases.	[179]

## 13. Application of Xylanolytic Yeasts

Xylan-degrading enzymes are applied in an array of industrial and commercial processes, from the paper and pulp industry to sweetener production [189]. They are supplemented in animal feed to improve the digestibility of nutrients [190]. The role of xylanase to bleach kraft pulp is, by far, the most common application of this enzyme [191]. Lately, xylanases have been applied for the synthesis of a natural sweetener, xylitol [192]. Owing to the high demand for xylanases in industrial processes, the exploration of xylanase-over-producing strains is a biotechnologically relevant topic. Bacterial and mold strains have largely been exploited in this regard. The genera *Bacillus*, *Trichoderma* and *Aspergillus* have widely been reported for their potential to produce higher titers of xylanases [187]. Yeasts have, however, not been reported for the commercial production of xylanase. The bleaching of kraft pulp is carried out at a higher temperature (70–100 °C) [110]; therefore, commercially available enzymes such as Cartazyme^®^, Ecopulp X200^®^ and Resinase^®^, which are thermolabile, cannot be employed for that purpose [193]. Consequently, the exploration of sources of thermostable xylanase producers is an ongoing task [194]. Reportedly, the cellulase-free xylanase from the yeast *A. pullulans* [195] is suitable for the pulp and paper industry, as it provides greater yield with minimum loss of cellulose. Moreover, xylanase from a color variant strain CBS135684 of *A. pullulans* [196] was found to be relatively thermostable. At higher temperatures, changes in enzyme conformation cause the loss of enzyme activity [197], which can sometimes be avoided by supplementing some additives such as polyols, which promote the formation of hydrogen bonds or salt bridges between amino acid residues and hence improve the thermal stability of the enzyme [198]. However, the same additive cannot enhance all the types of xylanases; few other additives have been reported.

Bankeeree et al. [176] purified the xylanase from the mentioned strain, *A. pullulans* CBS 135684, and assessed its prospects in the biobleaching of pulp. They used rice straw fibers to prepare the pulp instead of kraft pulp and found that xylanase pretreatment prior to H_2_O_2_ bleaching significantly increased the fiber brightness. Moreover, the positive impact of sorbitol on xylanase efficiency in the pulping process was also evident. A sequential treatment of xylanase and 10% (*v*/*v*) H_2_O_2_ to the pulp, in comparison to the treatment with H_2_O_2_ alone, increased the brightness of the prepared sheets by 13.5% and improved the tensile and tear strengths of the pulp by up to 1.16- and 1.71-fold, respectively.

Xylanases have also been reported for the hydrolysis of lignocellulosic biomass to obtain biofuels or other value-added products. Shariq and Sohail [172] demonstrated that the yeast strain *Candida tropicalis* MK-160 co-produces xylanase and endoglucanase; the enzyme preparation can be utilized for saccharification. In addition, the strain was able to produce ethanol on a sugar-containing medium.

Many yeast species have been reported to ferment xylose; however, very few ethanologenic yeasts can convert this xylose to ethanol. Further, the inhibitors released from plant biomass hydrolysate impede yeast growth [199,200]. Hence, the commercial production of ethanol from xylose has yet to be achieved. Therefore, the isolation and identification of yeast strains that can ferment hemicellulosic sugars, particularly D-xylose, will improve the prospects for lignocellulosic ethanol production [201].

The feasibility of application of xylanase in the preparation of xylooligosaccharides as prebiotics has also been studied. Ding et al. [184] applied xylanase from *Pichia stipitis* to obtain a xylan hydrolysate with a 92% xylooligosaccharide yield. Specifically, the hydrolysate contained 14% xylotetraose, 49% xylotiose and 29% xylobiose [184].

## 14. Conclusions and Future Prospects

Cellulases and xylanases obtained from yeasts are efficient catalysts with industrially relevant properties. Yet, the commercial exploitation of these yeasts requires further study, particularly for scaled-up production of the enzymes. This is particularly important as bulk production of cellulases and xylanases is still not economically feasible and is considered a major impediment in the development of lignocellulosic fuels. In this regard, the co-production of cellulase and xylanase using a single waste material can help to decrease the production cost.

## Figures and Tables

**Table 1:** Characteristics of several cellulose types.

Indicator, %	Types of Cellulose			Source
	SC	CC	HC	
α-cellulose	99.0	98.9	95.4	[20]
residual lignin	0.51	--	0.17	[20]
pentosans	0.17	--	--	[25]
ash	0.13	0.13	0.16	[26]

**Table 4:** Examples of reported xylanolytic yeasts.

Origin	Source	*Isolated from*	Strains	Reference
Terrestrial	Assiut region, Egypt	*-*	*Saccharomyces cerevisiae*	[10]
	Mushroom farm in Yala Local Government Area of Cross River State, Nigeria	Soil samples	*Saccharomyces cerevisiae* SCPW 17	[85]
	Brazil	Decaying wood and sugarcane bagasse	*Meyerozyma guilliermondii*, *Scheffersomyces shehatae*, *Sugiyamaella smithiae*	[168]
	Two Atlantic Rainforest habitats in Brazil	Environmental and food samples including garden soil, plant parts, grapes, lemon, green chili and orange juice	*Candida tropicalis* MK-160	[172]
	Icepack at the Antarctic station Dumont d’Urville (60°409 S; 40°019 E)	Decayed algae	*Cryptococcus adeliae*	[174]
	Four Atlantic Rainforest sites in Brazil	Samples of rotting wood	*Sugiyamaella xylanicola* and *Saitozyma podzolica*, *Apioctrichum sporotrichoides*, *Aureobasidium pullulans*	[177]
	Different cities in southern Rio Grande do Sul	Chicory	*Cryptococcus laurentii*	[178]
	Agharkar Research Institute, Pune, Maharashtra (18.5207451° N, 73.8315643° E), and Singalandhapuram, Namakkal District, Tamil Nadu (11.420428° N, 78.220487° E)	Wood-feeding termites	*Pseudozyma hubeiensis*, *Hannaella pagnoccae*, *Papiliotrema mangalensis*, *Kodamaea ohmeri* TS21	[179]
	Rotting wood trees at Huazhong Agricultural University, Wuhan, China	Wood-feeding termite *Reticulitermes chinensis*	*Barnettozyma californica*, *Candida* sp., *Cyberlindnera* sp., *Sterigmatomyces halophilus*, *Sugiyamaella smithiae*, *Vanrija humicola* and *Wickerhamomyces* sp.	[181]
	Two Atlantic Rainforest habitats in Brazil	Rotting wood	*Spencermartinsiella* sp. 1, *Sugiyamaella xylanicola*, *Tremella* sp.	[182]
	Assiut region, Egypt	Soil samples	*Pichia membranifaciens*	[183]
	-	Soil samples	*Pichia stipitis*	[184]
	Thailand	Leaves, painted wall surfaces and wood surfaces	*Aureobasidium pullulans* CBS 135684	[185]
	Brazil	Rotting sugarcane bagasse and wood samples	*Sugiyamaella* sp.	[186]
Marine	Antarctic	Sea squirt	*Candida* *davisiana*	[180]

## Data Availability

Not applicable.
